# SAMHD1 Phosphorylation at T592 Regulates Cellular Localization and S-phase Progression

**DOI:** 10.3389/fmolb.2021.724870

**Published:** 2021-08-26

**Authors:** Stephanie Batalis, LeAnn C. Rogers, Wayne O. Hemphill, Christopher H. Mauney, David A. Ornelles, Thomas Hollis

**Affiliations:** ^1^ Department of Biochemistry, Wake Forest School of Medicine, Winston-Salem, NC, United States; ^2^ Department of Microbiology and Immunology, Wake Forest School of Medicine, Winston-Salem, NC, United States

**Keywords:** SAMHD1, phosphorylation, protein localization, protein oxidation, cell cycle, dNTP

## Abstract

SAMHD1 activity is regulated by a network of mechanisms including phosphorylation, oxidation, oligomerization, and others. Significant questions remain about the effects of phosphorylation on SAMHD1 function and activity. We investigated the effects of a SAMHD1 T592E phosphorylation mimic on its cellular localization, catalytic activity, and cell cycle progression. We found that the SAMHD1 T592E is a catalytically active enzyme that is inhibited by protein oxidation. SAMHD1 T592E is retained in the nucleus at higher levels than the wild-type protein during growth factor-mediated signaling. This nuclear localization protects SAMHD1 from oxidation by cytoplasmic reactive oxygen species. The SAMHD1 T592E phosphomimetic further inhibits the cell cycle S/G2 transition. This has significant implications for SAMHD1 function in regulating innate immunity, antiviral response and DNA replication.

## Introduction

SAMHD1 is a dNTP triphosphohydrolase (dNTPase) that hydrolyzes the alpha linkage of dNTPs (Goldstone et al., 2011; Powell et al., 2011). It has emerged as a central component of several critical biological functions that depend on dNTP regulation, such as DNA replication and repair, cell cycle progression, and regulation of the innate immune response (Mauney and Hollis, 2018). Its importance is underscored by the fact that mutations in the SAMHD1 gene lead to the severe autoimmune disease Aicardi-Goutieres syndrome (AGS) and have been identified as a contributing factor to several cancers, including chronic lymphocytic leukemia (CLL), colon cancer, and lung cancer (Mauney and Hollis, 2018). SAMHD1 is also a viral restriction factor that suppresses retroviruses such as HIV-1, and several DNA viruses by lowering cellular dNTP concentrations needed for viral reverse transcription or DNA replication (Berger et al., 2011; Goldstone et al., 2011; Hrecka et al., 2011; Laguette et al., 2011; Powell et al., 2011; Lahouassa et al., 2012; Gramberg et al., 2013; Hollenbaugh et al., 2013; Kim et al., 2013, 2019; Sze et al., 2013; Chen et al., 2014; Pauls et al., 2014; Wittmann et al., 2015; Sommer et al., 2016; Businger et al., 2019). In order to coordinate its diverse biological roles, SAMHD1 activity is regulated by several orthogonal mechanisms including protein tetramerization, phosphorylation, oxidation, acetylation, and sumoylation (Hofmann et al., 2012; White TE. et al., 2013; Cribier et al., 2013; Ji et al., 2013; Welbourn et al., 2013; Lee et al., 2017; Mauney et al., 2017; Wang et al., 2018; Martinat et al., 2020).

SAMHD1 is phosphorylated at residue threonine 592 (T592) by CDK1/2 and cyclin A (White TE. et al., 2013; Cribier et al., 2013; Pauls et al., 2014; St.; Yan et al., 2015; Gelais et al., 2016) and dephosphorylated by phosphatase PP2A in a cell cycle dependent fashion (Schott et al., 2018; Tramentozzi et al., 2018). Phosphorylated SAMHD1 fails to restrict HIV replication (White TE. et al., 2013; Cribier et al., 2013; Welbourn et al., 2013; Wittmann et al., 2015; Bhattacharya et al., 2016). The effects of phosphorylation on catalytic activity are still under debate, however. Some evidence suggests phosphorylation negatively modulates SAMHD1 dNTPase activity, and correlates SAMHD1 phosphorylation with elevated intracellular dNTP pools (Pauls et al., 2014; Arnold et al., 2015; Ruiz et al., 2015; Tang et al., 2015; Wittmann et al., 2015; Yan et al., 2015). Other studies find no effect of phosphorylation of SAMHD1 on catalytic activity (White TE. et al., 2013; Welbourn et al., 2013; Bhattacharya et al., 2016; Tramentozzi et al., 2018).

SAMHD1 catalytic activity is inhibited by oxidation during growth factor-mediated signaling by reactive oxygen species (ROS) (Mauney et al., 2017; Wang et al., 2018). It is now generally established that ROS play a critical role in transmitting cell signals in response to growth factors, including lysophosphatidic acid (LPA) (Chen et al., 1995; Sundaresan et al., 1995; Bae et al., 1997; Oakley et al., 2009; Saunders et al., 2010; Klomsiri et al., 2014). SAMHD1 contains three cysteine residues that sense ROS and participate in a “redox switch” resulting in reversible catalytic inhibition (Mauney et al., 2017; Wang et al., 2018). Additionally, growth factor stimulation by LPA causes SAMHD1 translocation from the nucleus to the cytoplasm where it accumulates in its oxidized form (Mauney et al., 2017).

SAMHD1 is a nuclear protein directed by a nuclear localization sequence (NLS) on the N-terminus (Brandariz-Nuñez et al., 2012; Hofmann et al., 2012; Wei et al., 2012; Schaller et al., 2014). However, evidence now suggests that SAMHD1 subcellular localization is closely linked to protein function. Translocation from the nucleus to the cytoplasm has been observed as a response to growth factor stimulation and also a mechanism to suppress LINE-1 retroelements (Mauney et al., 2017; Du et al., 2019). SAMHD1 subcellular localization is further linked to retroviral restriction through its degradation by the HIV-2 and simian immunodeficiency virus (SIV) nuclear accessory protein Vpx (Berger et al., 2011; Hrecka et al., 2011; Laguette et al., 2011). Vpx relies on a nuclear E3 ubiquitin ligase to target SAMHD1 for proteasomal degradation; cytoplasmic SAMHD1 does not interact with the nuclear machinery and is protected from this degradation pathway (Brandariz-Nuñez et al., 2012; Hofmann et al., 2012; Wei et al., 2012; Schaller et al., 2014). SAMHD1 that is localized to the cytoplasm by deletion of the NLS is fully capable of restricting HIV-1 infection (Brandariz-Nuñez et al., 2012; White T. E. et al., 2013; Schaller et al., 2014).

Significant open questions remain regarding SAMHD1 regulation by post-translational modifications, such as how they affect catalytic activity and cellular localization, as well as if they are interdependent on one another. Here we show that SAMHD1 phosphorylation directs protein localization in the cellular response to LPA stimulation. Phosphorylation prevents LPA-induced SAMHD1 translocation from the nucleus and the resulting cytoplasmic oxidation, preserving catalytic activity. As a catalytically active and nuclear dNTPase, phosphorylated SAMHD1 prevents normal S-phase progression and exit. Our results demonstrate the interdependence of SAMHD1 phosphorylation, cellular localization, and oxidation, and the subsequent downstream effects on cell cycle dynamics.

## Results

### SAMHD1 T592E Phosphomimetic does not Translocate Out of the Nucleus After Growth Factor Stimulation

Given that SAMHD1 has been previously shown to translocate from the nucleus in response to growth factor signaling, and that post-translational modifications can be involved in protein trafficking, we tested the potential effects of T592 phosphorylation on SAMHD1 cellular localization. We expressed YFP-tagged variants of SAMHD1 including the phosphomimetic (T592E), phospho-insensitive (T592A), or wild-type SAMHD1 in unsynchronized HEK293 and PC3 cell lines and determined SAMHD1 localization by fluorescence microscopy. The T592E variant has been used extensively as a phosphomimetic to investigate its effects on SAMHD1 function and activity (White T. E. et al., 2013; Cribier et al., 2013; Welbourn et al., 2013; Bhattacharya et al., 2016; Coquel et al., 2018). These experiments were done in PC3 cells, in which we previously showed that SAMHD1 translocates to the cytoplasm in response to LPA treatment, and HEK293 cells as an additional non-cancerous cell line (Mauney et al., 2017).

**FIGURE 1:**
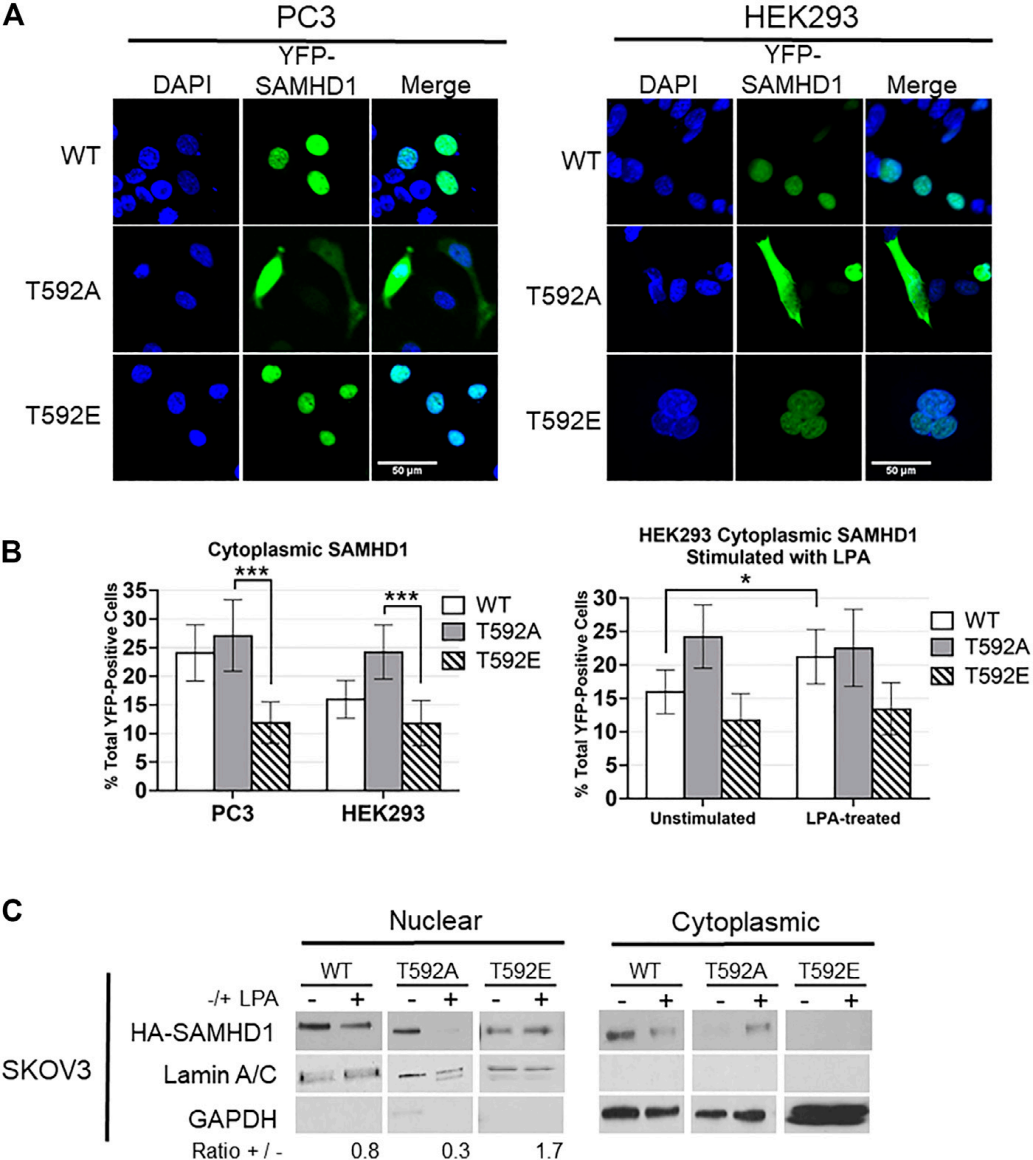
SAMHD1 phosphorylation affects localization. **(A)** Fluorescent confocal microscopy of PC3 and HEK293 cells expressing YFP-SAMHD1 (green) with DAPI stain (blue). **(B)** Percentage of YFP-positive cells with cytoplasmic SAMHD1. Error bars represent 95% highest density intervals. *p < 0.05, ***p < 0.001, Fisher’s exact test. A minimum of 200 cells per time point were counted from 3 (HEK293) or 4 (PC3) independent experiments. **(C)** Cytoplasmic and nuclear fractionation of SKOV3 cells expressing HA-SAMHD1. Lamin A/C and GAPDH serve as loading and fractionation controls. Densitometry was performed to determine the ratio of post-treatment to pre-treatment nuclear SAMHD1 after normalizing to the Lamin A/C loading control. Blot is representative of three independent experiments.

Only about 12% of cells expressing the fluorescent T592E SAMHD1 phosphomimetic showed localization of the protein in the cytoplasm in both PC3 and HEK293 unstimulated cells (Figures 1A,B). The phosphorylation-insensitive T592A SAMHD1 was localized outside of the nucleus approximately two-fold more often than T592E SAMHD1 (PC3 *p* = 2.0586e-05, HEK293 *p* = 0.000124, Fisher’s exact test) (Figures 1A,B). Wild-type SAMHD1, which is regulated by endogenous cellular mechanisms and is likely a mixture of phosphorylated and unphosphorylated protein, displayed an intermediate level of localization to the cytoplasm compared to T592A or T592E SAMHD1. Stimulation of HEK293 cells expressing SAMHD1 variants with the growth factor LPA resulted in an increase of WT SAMHD1 in the cytoplasm after 10 min (*p* = 0.049855, Fisher’s exact test) (Figure 1B). In contrast, T592E SAMHD1 did not increase in the cytoplasm after treatment, nor did the T592A SAMHD1, which already had increased localization to the cytoplasm. This analysis does not measure changes to the fluorescence intensity in the cytoplasm post-stimulation and only takes into consideration the number of cells containing cytoplasmic SAMHD1 (Figure 1B).

Using cell fractionation, we also observed that T592E SAMHD1 remained nuclear following treatment with LPA in SKOV3 cells (Figure 1C). SKOV3 cells were chosen as they respond to LPA stimulation by generating ROS (Saunders et al., 2010; Klomsiri et al., 2014; Rogers et al., 2018). Cells stably expressing N-terminal HA-tagged SAMHD1 mutants were stimulated or not with LPA and fractionated into cytoplasmic and nuclear fractions. The nuclear fraction of both WT and T592A SAMHD1 decreased following stimulation, which in contrast to T592E SAMHD1 did not decrease following LPA treatment.

### T592 Mutations Affect SAMHD1 Oxidation After Treatment with Growth Factor

Many growth factor signaling pathways, including LPA signaling, transmit signals by generating ROS to oxidize downstream target proteins (Chen et al., 1995; Sundaresan et al., 1995; Bae et al., 1997; Oakley et al., 2009; Saunders et al., 2010; Klomsiri et al., 2014). We previously showed that growth factor stimulation causes cytoplasmic localization and oxidation of SAMHD1 (Mauney et al., 2017). In light of the result that T592 mutations affect SAMHD1 localization, we hypothesized that preventing translocation to the cytoplasm also inhibits growth factor-mediated oxidation in the cytoplasm. We next tested whether the SAMHD1 variants could be oxidized in response to LPA stimulation. To label oxidized proteins, we used the dimedone-based probe DCP-Bio1 that forms a covalent adduct with cysteine sulfenic acid, an intermediate of cysteine oxidation (Nelson et al., 2010).

**FIGURE 2:**
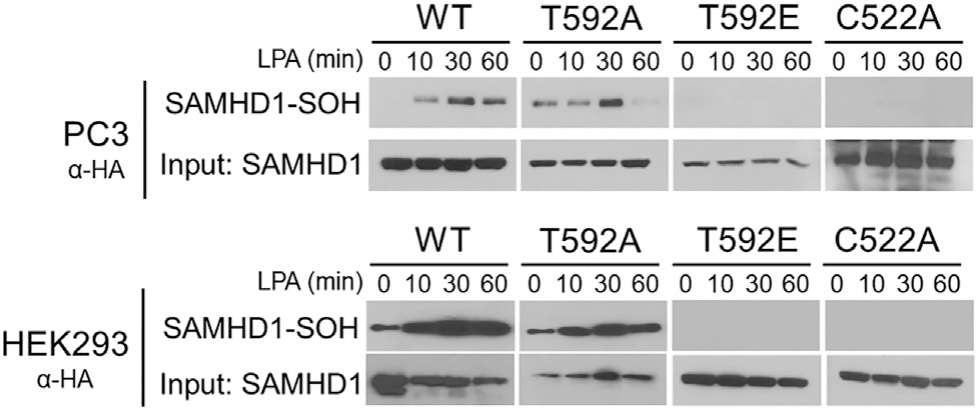
SAMHD1 T592E is not oxidized in cells. PC3 and HEK293 cells expressing HA-SAMHD1 were treated with the growth factor, LPA, over time and harvested in the presence of DCP-Bio1 to capture proteins with cysteine sulfenic acids. Western blots of pulldowns probed with α-HA antibodies show oxidized SAMHD1 in cells expressing wild-type or T592A variant. No oxidized SAMHD1 appears in cells expressing the T952E or oxidation insensitive C522A variants.

PC3 and HEK293 cells transiently expressing HA-tagged variants of SAMHD1 (WT, T592A, or T592E) were treated with LPA and harvested in the presence of DCP-Bio1 to label oxidized proteins (Mauney et al., 2017). Labeled proteins were purified by affinity capture of the biotin tag and separated by SDS-PAGE. Immunoblotting for the HA-tag allowed visualization of oxidized SAMHD1 (Nelson et al., 2010; Mauney et al., 2017). Oxidation of the wild-type SAMHD1 and T592A-SAMHD1 proteins was detected within 30 min of growth factor treatment (Figure 2). In contrast, no oxidation of the T592E SAMHD1 was detected by the probe after stimulation with growth factor. As a control, the C522A SAMHD1 protein that is oxidation-insensitive was also not detected by the probe post growth-factor treatment (Figure 2). Taken together, these results suggest that the phosphomimetic SAMHD1 mutant affects both protein translocation and oxidation in response to growth factor stimulation.

### Phosphomimetic SAMHD1 is Catalytically Active and Inhibited by Oxidation

**FIGURE 3:**
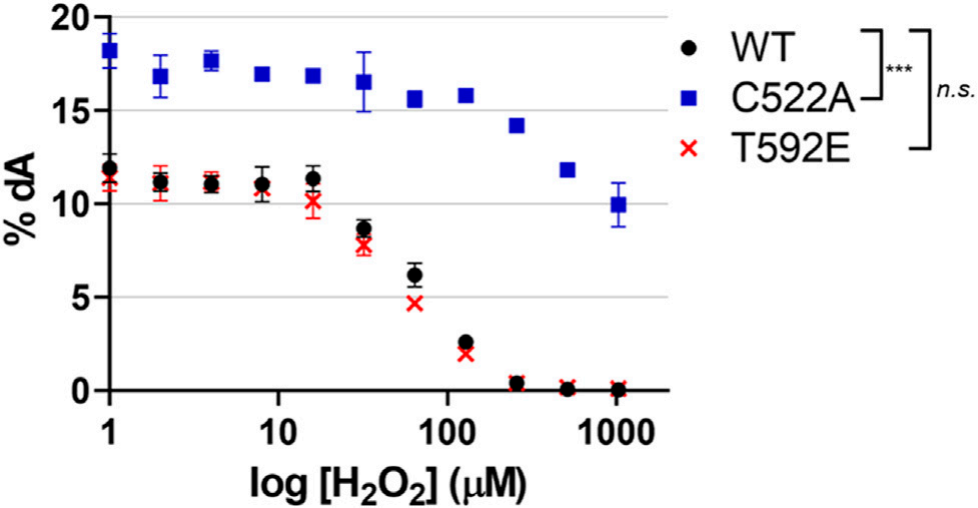
SAMHD1 T592E is catalytically active and sensitive to oxidation. Catalytic activity was of SAMHD1 wild-type, T592E, and C522A were measured *in vitro* as a function of increasing hydrogen peroxide concentrations. The T592E variant shows nearly identical activity and sensitivity to peroxide inhibition as wild-type protein. As a control, the C522A SAMHD1, which is insensitive to oxidation, remains active even at high concentrations of peroxide. Activity was determined by the percentage of dATP that was converted to the product dA. Hydrogen peroxide concentration is presented on a logarithmic scale. The mean of three independent experiments are plotted, error bars represent standard error of the mean (SEM). Data were normalized to WT at 0 µM H_2_O_2_ for analysis and *p*-values between genotypes were generated by Tukey-Kramer post-hoc test. n. s. *p* > 0.5 at each time point comparing WT and T592E, ***p < 0.001 at each time point comparing WT and C522A.

Taking into consideration the lack of oxidized T592E in cells, we next wanted to determine if the absence of the T592E oxidation in cells was an artifact of the mutation that might have disturbed protein structure or activity. We hypothesized that the absence of T592E oxidation was due to protein localization rather than a biochemical inability to be oxidized. To determine if T592E SAMHD1 is catalytically active and susceptible to inactivation by H_2_O_2_, we measured the hydrolysis of dNTP substrates by SAMHD1 proteins in the presence of increasing concentrations of hydrogen peroxide *in vitro* using an HPLC-based assay (Mauney et al., 2017). In the absence of hydrogen peroxide the wild-type, T592E, and C522A SAMHD1 proteins hydrolyze dATP to dA with similar activity (Figure 3). Consistent with previous data, C522A SAMHD1 exhibits higher baseline levels of catalytic activity, likely due to its resistance to low levels of contaminating oxidants (Mauney et al., 2017). Upon addition of increasing concentrations of hydrogen peroxide, the catalytic activity of wild-type and T592E SAMHD1 proteins decreases while the oxidation-insensitive C522A mutant retains catalytic activity (Figure 3). This result confirms that T592E SAMHD1 is both catalytically active and sensitive to inhibition by oxidation. It also indicates that the lack of oxidized T592E SAMHD1 in response to growth factor treatment in the cellular assay is not due to a biochemical insensitivity to oxidation.

### Phosphomimetic SAMHD1 Affects Cell Proliferation

Given the role of SAMHD1 as a dNTPase that regulates dNTP availability during DNA replication, we hypothesized that phosphorylated SAMHD1, which remains in the nucleus where it is protected from inactivation by oxidation, would inhibit S-phase progression and cell cycle dynamics.

**FIGURE 4:**
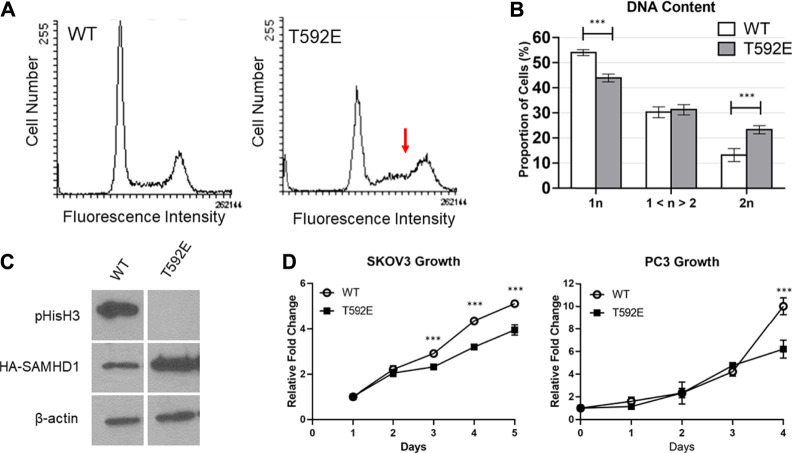
SAMHD1 T592E affects cell cycle progression. **(A)** DNA histogram of PC3 cells stably expressing WT or T592E SAMHD1 (10,000 events per histogram). Cells expressing wild-type SAMHD1 show a typical cell cycle distribution. In contrast, cells expressing T592E SAMHD1 indicate an accumulation in late S-phase (red arrow). Graphs are representative of three independent experiments. **(B)** Quantification of DNA content by flow cytometry. The proportion of cells in each phase was determined by the Dean-Jett-Fox model. Graphs represent the mean of WT n = 14 and T592E n = 15 from three independent experiments. Error bars represent 95% confidence intervals. *p < 0.05, ***p < 0.001, Tukey-Kramer post-hoc test. **(C)** Expression of phosphorylated Histone H3 can be detected in PC3 cells stably expressing wild-type SAMHD1, but not T592E SAMHD1, suggesting fewer cells expressing T592E are undergoing mitosis. Expression of HA-SAMHD1 was probed by anti-HA antibody. Samples are from the same blot. **(D)** Cells stably expressing wild-type SAMHD1 grow faster than those expressing T592E SAMHD1. Relative fold change of SKOV3 cells was measured by SRB stain assay. Points represent the mean and error bars represent the standard deviation of 10 technical replicates. Relative fold change of PC3 cells was measured by counting with trypan blue. Points represent the mean and error bars represent the standard deviation of three technical replicates. *p < 0.05, ***p < 0.001, Tukey-Kramer post-hoc test.

We measured the DNA content of unsynchronized PC3 cells expressing wild-type or T592E SAMHD1 by flow cytometry to analyze cell cycle distribution. Cells expressing wild-type SAMHD1 displayed a typical profile of DNA content that remains consistent throughout S-phase and has a clear definition between S-phase and G2/M (Figure 4). In contrast, cells expressing the T592E SAMHD1 displayed an upward slope of cells in S-phase that merged into the 2n DNA peak (Figures 4A,B), suggesting an accumulation in late S-phase or early G2/M-phase of the cell cycle. Quantification of the area under these peaks reveals a statistical significance between the number of cells having 1n and 2n DNA (Figure 4B). Given the unusual accumulation of cells in late S-phase merging into the 2n DNA peak, we looked to see whether the cells expressing T592E were entering mitosis. We measured the level of mitosis-related protein phospho-Histone H3, which is present during late G2 and early M-phase and indicates the presence of cells that have successfully completed S-phase (Figure 4C). Phospho-Histone H3 was present in PC3 cells expressing wild-type but not T592E SAMHD1. Taken together, these results indicate that phosphomimetic SAMHD1 causes inefficient progression through S-phase and defective exit from S-phase into mitosis.

To determine whether the accumulation of cells in late S-phase is accompanied by proliferative differences, we measured the growth rate of cells stably expressing either wild-type or T592E SAMHD1 (Figure 4D). Expression of T592E SAMHD1 decreased the growth rate of PC3 and SKOV3 cells (PC3 *p* = 5.73e-07, SKOV3 *p* = 4.35e-10 by day 5, two-way ANOVA and Tukey’s post-hoc test). This result indicates that the accumulation of cells expressing phosphomimetic SAMHD1 at the S/G2 transition is associated with decreased proliferation.

## Discussion

Here, we demonstrate a link between SAMHD1 phosphorylation, cellular localization, and protein oxidation. We observe that most of the T592E phosphomimetic protein is retained in the nucleus following LPA-mediated signaling, whereas a significantly increased proportion of the non-phosphorylated protein localizes to the cytoplasm. We also show that the T592E protein fails to become oxidized in cells in response to LPA treatment. Additionally, our data reveal that phosphomimetic T592E SAMHD1 is an active dNTPase that is sensitive to inhibition by oxidation *in vitro*. Our results further indicate that phosphorylation retains SAMHD1 in the nucleus where it inhibits S/G2 transition and decreases cell proliferation.

While SAMHD1 is considered a nuclear protein (Brandariz-Nuñez et al., 2012; Goncalves et al., 2012; Hofmann et al., 2012; Guo et al., 2013), there is increasing evidence pointing to the importance of SAMHD1 translocation to the cytoplasm for its function (Herrmann et al., 2018; Du et al., 2019). For example, nucleocytoplasmic shuttling of SAMHD1 is necessary for LINE-1 retroelement (L1) suppression, and mutants that fail to suppress L1 replication cannot export out of the nucleus (Du et al., 2019). Other work finds that expression of SAMHD1 T592A strongly inhibits L1 retrotransposition, while the T592D phosphomimetic does not (Herrmann et al., 2018). Our finding that phosphorylation regulates SAMHD1 nuclear localization unifies these data and supports the mechanism of phosphorylation of SAMHD1 regulating L1 suppression.

Linking SAMHD1 nucleocytoplasmic shuttling to phosphorylation also provides a potential explanation for the loss of retroviral restriction upon SAMHD1 phosphorylation. SAMHD1 restricts retroviruses at the level of reverse transcription (Laguette et al., 2011; Lahouassa et al., 2012; Gramberg et al., 2013; Sze et al., 2013; Sommer et al., 2016), and SAMHD1 localized to the cytoplasm through mutation or deletion of its NLS is capable of restricting retroviruses (Brandariz-Nuñez et al., 2012; Schaller et al., 2014). Phosphorylation of SAMHD1 at T592 ablates SAMHD1 viral restriction (White T. E. et al., 2013; Cribier et al., 2013; Welbourn et al., 2013), but does not affect its catalytic activity as we and others have observed (White T. E. et al., 2013; Welbourn et al., 2013; Bhattacharya et al., 2016; Tramentozzi et al., 2018), suggesting there is another mechanism of inactivation. Our finding that phosphorylated SAMHD1 is retained in the nucleus, away from reverse transcription in the cytoplasm, is a potential explanation for this phenomenon.

SAMHD1 has defined nuclear roles as well, including in DNA repair and maintenance of dNTP levels for DNA replication. These nuclear roles are fundamentally coupled to cell cycle dynamics, as DNA replication and repair by homologous recombination both occur during S-phase. SAMHD1 is phosphorylated during S-phase by Cyclin A2/CDK1 and dephosphorylated by PP2A during M-phase (Cribier et al., 2013; Cribier et al., 2013; White TE. et al., 2013; Pauls et al., 2014; Yan et al., 2015; Gelais et al., 2016; Schott et al., 2018; St.). The T592E SAMHD1 phosphomimetic has enhanced nuclear DNA-repair function at stalled replication forks compared to phosphorylation-insensitive protein (Coquel et al., 2018) and phosphorylated SAMHD1 increases after treatment with agents that induce DNA double-strand breaks (Clifford et al., 2014). Consistent with this DNA repair function and our observation that phosphomimetic SAMHD1 slows S-phase progression, phosphorylated SAMHD1 may retain the enzyme in the nucleus for DNA repair and function as part of a check point to prevent cell cycle progression until proper DNA replication or repair is completed.

**FIGURE 5:**
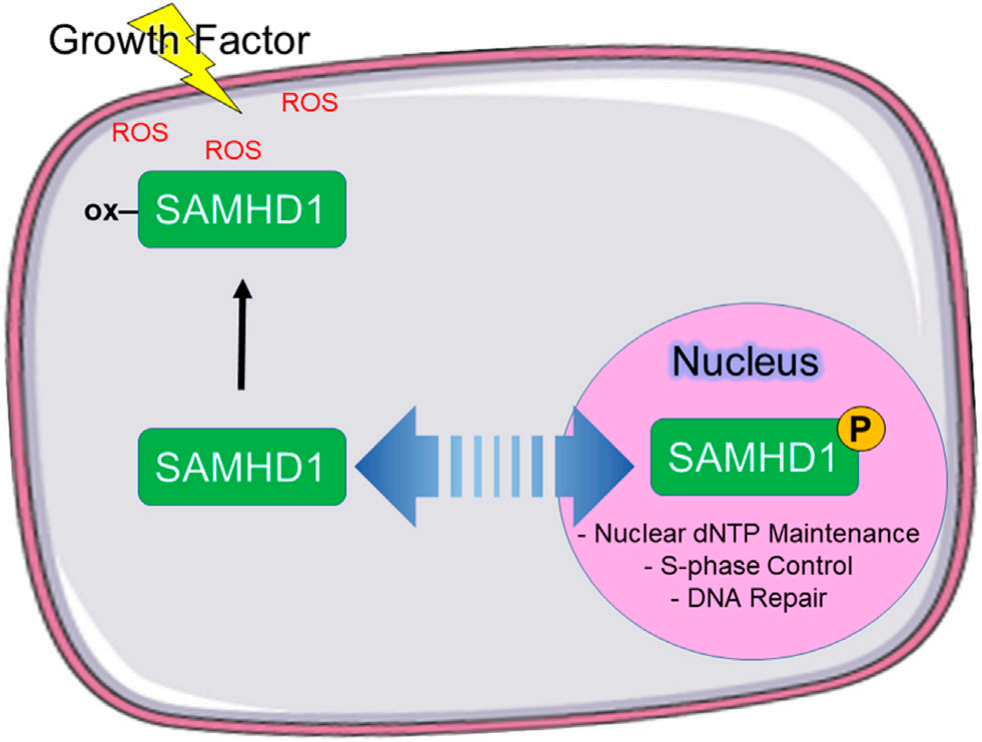
Proposed model of SAMHD1 localization and cellular function. We propose that phosphorylation directs subcellular localization, which in turn directs cellular function. In our model, phosphorylated SAMHD1 is retained in the nucleus where it is able to participate in nuclear functions. Unphosphorylated SAMHD1 can be stimulated to leave the nucleus, where it can be oxidized in response to growth factor-stimulated ROS.

Based on these data, we propose a model in which phosphorylation of SAMHD1 spatiotemporally regulates protein function (Figure 5). We propose that SAMHD1 localization is in an equilibrium between nuclear import and export with the balance dictated by the particular needs of the cell for regulating innate immunity, antiviral response, and DNA replication. The NLS controls nuclear import and the phosphorylation state, in part, controls export. In this model, non-phosphorylated SAMHD1 can be shuttled to the cytoplasm for immune regulation such as L1 suppression. Under conditions of growth factor stimulation, cytoplasmic localization also allows further regulation of SAMHD1 catalytic activity by protein oxidation. Retroviral restriction may also be dependent on SAMHD1 localization to the cytoplasm.

The relationship between SAMHD1, dNTP levels, and cell cycle regulation is complex and not yet fully understood. This complexity is reflected in SAMHD1 regulation by a network of orthogonal post-translational modifications including phosphorylation, oxidation, oligomerization, acetylation, and sumoylation (Lee et al., 2017; Mauney and Hollis, 2018; Martinat et al., 2020). These modifications are interdependent, as we show here in the context of protein phosphorylation and oxidation. Our data underscore the need to consider these orthogonal modifications in the context of one another to unravel the dynamic network of SAMHD1 regulation.

## Materials and Methods

### Reagents

RPMI, DMEM, and FBS were from Invitrogen. Hygromycin B and puromycin were from Gibco. Primary antibodies used were anti-HA (Cell Signaling Technology, catalog number C29F4), anti-pHistoneH3 (S10) (Abcam, catalog number ab14955), anti-LaminA/C (BD Biosciences, catalog number 612162), anti-GAPDH (Sigma-Aldrich, catalog number CB1001), and anti-β-actin (Santa Cruz Biotechnology, Inc., catalog number sc-1616). Secondary HRP-conjugated antibodies used were goat anti-rabbit (Cell Signaling Technology, catalog number 7074S) horse anti-mouse (Cell Signaling Technology, catalog number 7076S) and donkey anti-goat (Santa Cruz Biotechnology, Inc., catalog number sc-2020). Chemiluminescence reagent for Western blotting was SuperSignal™ West Pico PLUS. Mounting media for fluorescence microscopy was ProLong® Gold Antifade Reagent with DAPI. LPA supplied in chloroform was from Avanti Polar Lipids {Acyl-linked 18:1 lysophosphatidic acid [1-oleoyl-2-hydroxy-sn-glycero-3-phosphate (sodium salt)]}. For sulfenylation studies, DCP-Bio1 was from by Xoder Technologies (Winston Salem, NC), sepharose CL-4B resin was from Sigma, and high capacity streptavidin agarose resin was from Thermo Scientific. Propidium iodide and the nucleotides GTP and dATP were from Invitrogen.

### Plasmids

For bacterial expression, the full-length human SAMHD1 gene was amplified using PCR and cloned into a modified pET28 expression vector (pLM303-SAMHD1) that contained an N-terminal his-MBP tag and an intervening rhinovirus 3C protease cleavage site. Threonine to alanine (T592A), threonine to glutamic acid (T592E), and cysteine to alanine (C522A) mutations were synthesized and sequenced by GenScript, using the pLM303-SAMHD1 expression vector as a template. For mammalian expression, the full-length human SAMHD1 gene with an N-terminal HA-tag was cloned into the pcDNA3.1 (+) Hygro plasmid. T592A, T592E, and C522A mutations were synthesized and sequenced by GenScript. For fluorescence microscopy SAMHD1 mutants were cloned from pcDNA3.1 (+)Hygro into pEYFP-C1 (Clontech) by GenScript.

### Cell Culture and LPA Treatment

PC3 cells (ATCC) and HEK293 cells (ATCC) were grown, maintained, and treated at 37°C with 5% CO_2._ PC3 cells were maintained in RPMI 1640 medium supplemented with 10% fetal bovine serum, l-glutamine, penicillin, and streptomycin. Where indicated, serum-starvation was conducted for 36 h with RPMI 1640 serum-free medium supplemented with l-glutamine, penicillin, and streptomycin. HEK293 cells were maintained in DMEM medium supplemented with 10% fetal bovine serum, l-glutamine, penicillin, and streptomycin. Transfection into PC3 or HEK293 cells was performed with 2 µg of plasmid DNA, Opti-MEM reduced serum medium, and FuGENE® six Transfection Reagent per the manufacturer’s instructions. Cells stably expressing transfected constructs were selected with Hygromycin B at 400 μg/ml. LPA, supplied in chloroform, was dried under a stream of argon, resuspended to a concentration of 1 mM in phosphate buffered saline (PBS) containing 1% fatty acid-free bovine serum albumin (BSA), and then diluted into culture medium to a final concentration of 1 µM.

### Cell Growth

SKOV3 cells stably expressing WT or T592E SAMHD1 were plated at 4,000 cells per well in 96-well plates and allowed to grow for the indicated number of days. Cells were fixed in 50% trichloroacetic acid for 1 h at 4°C. Cells were rinsed 5 times with deionized water and allowed to dry completely. Cells were incubated in 0.4% sulforhodamine B sodium salt (Sigma) in 1% acetic acid for 10 min to stain and washed 5 times with 1% acetic acid. Dye was solubilized in 10 mM non-pH adjusted Tris-base and absorbance was measured at 564 nm. PC3 cells stably expressing WT or T592E SAMHD1 were plated at 75,000 cells per 35 mm dish and allowed to grow for the indicated number of days. Cells were removed from the plates with trypsin. Live cells were distinguished from dead cells by the addition of 100 µL of 0.4% trypan blue solution (Sigma) per 900 µL of cells. Cells were counted on a glass hemocytometer. For both methods, statistical analysis was performed by two-way ANOVA and significance was determined by Tukey-Kramer post-hoc test using RStudio (R Version 4.0.2, RStudio Version 1.1.453) (RStudio Team, 2020).

### Western Blotting

For Western blotting, cells were plated at 2.5 × 10^5^ cells per dish in 35-mm dishes, treated or not with LPA, washed with cold, calcium-free PBS, scraped into lysis buffer (50 mM Tris-HCl, 100 mM NaCl, 2 mM EDTA, 0.1% SDS, 0.5% sodium deoxycholate, 1 mM PMSF, 10 μg/ml aprotinin, 10 μg/ml leupeptin, 50 mM NaF, and 1 mM sodium vanadate), and centrifuged to remove cell debris after one freeze/thaw cycle. Protein concentration was measured (Pierce BCA protein assay) and samples (typically 20 μg protein/lane) were resolved on SDS polyacrylamide gels, then transferred to nitrocellulose membranes, probed with protein-specific antibodies and visualized using SuperSignal™ West Pico PLUS chemiluminescence reagent. Densitometry was analyzed by ImageJ software.

### Fluorescence Microscopy

PC3 or HEK293 cells were transiently transfected with YFP-SAMHD1 mutants as described above and allowed to rest for 24 h. Cells (∼5 × 10^4^ cells per well) were plated in 4-well chamber slides with removable wells and allowed to grow for 48 h before stimulation with 1 µM LPA (Nunc™ Lab-Tek™ II Chamber Slide™ System). Cells were then fixed for 15 min with 10% formalin, washed 3 times with wash buffer (2% FBS, 0.1 M glycine, PBS-T), permeabilized for 15 min with 0.1% Triton-X100 in PBS-T, and washed 3 times with PBS-T. After removing the chamber wells, cells were mounted under coverslips with ProLong® Gold Antifade Reagent with DAPI. Images were collected with an Olympus FV1200 Spectral Laser Scanning Confocal Microscope with an Olympus IX83 inverted platform. Identifying labels were removed from the images and given to two independent researchers to count the number of fluorescent cells and the number of fluorescent cells with cytoplasmic SAMHD1 per frame. A minimum of 200 cells per time point were counted from 3 (HEK293) or 4 (PC3) independent experiments. Statistical analysis was performed by Fisher’s exact test using RStudio (R Version 4.0.2, RStudio Version 1.1.453) (RStudio Team, 2020).

### Cytoplasmic/Nuclear Fractionation

Cells stably expressing WT, T592A, or T592E SAMHD1 were plated in 100 mm dishes and stimulated with 1 µM LPA as described. To harvest, cells were scraped into 300 µL of Lysis Buffer I (10 mM Tris-HCl pH 7.4, 10 mM NaCl, 3 mM MgCl_2_, 0.1 mM DTPA, 0.05% NP-40, 0.5% sodium deoxycholate, 2 mM DTT, 20 mM β-glycerophosphate, 1 mM PMSF, 10 μg/ml aprotinin, 10 μg/ml leupeptin, 50 mM NaF, and 1 mM sodium vanadate). Cells were incubated on ice for 10 min to lyse the cytoplasmic membrane and centrifuged (2 min, 10,000xg). The supernatant was collected as the cytoplasmic fraction and the pellet contained the cell nuclei. The pellet was washed with PBS and resuspended in 100 µL Lysis Buffer IV (20 mM Tris-HCl pH 7.4, 300 mM NaCl, 5 mM MgCl_2_, 0.2 mM DTPA, 1% Triton X-100, 1% SDS, 0.5% sodium deoxycholate, 2 mM DTT, 20 mM β-glycerophosphate, 1 mM PMSF, 10 μg/ml aprotinin, 10 μg/ml leupeptin, 50 mM NaF, and 1 mM sodium vanadate). Samples were sonicated to lyse the nuclear membrane and centrifuged (10 min, 10,000xg). The supernatant containing nuclear proteins was transferred to a new tube. Protein concentrations were determined by BCA assay.

### DCP-Bio1 Labelling and Affinity Capture

Labeling of cysteine sulfenic acids was performed as previously described with slight modifications (Klomsiri et al., 2014; Mauney et al., 2017). Briefly, PC3 or HEK293 cells (∼5 × 10^5^) transiently expressing HA-SAMHD1 mutants were grown in 100 mm plates for 48 h and treated with LPA for the indicated time points as described above. Cells were scraped into freshly prepared lysis buffer containing DCP-Bio1 (50 mM Tris-HCl, 100 mM NaCl, 0.1% SDS, 0.5% sodium deoxycholate, 1 mM PMSF, 50 mM sodium fluoride, 10 mM sodium vanadate, 10 μg/ml aprotinin, 10 μg/ml leupeptin, 200 U/mL catalase, 1 mM DCP-Bio1, 10 mM N-ethylmaleimide, 10 mM iodoacetamide). Harvested cells were incubated on ice for 30 min to chemically label cysteine sulfenic acids and were immediately stored at −80°C. For affinity capture and elution of the labeled proteins, samples were thawed, centrifuged to clear cell debris, and cleared using a BioGel P6 spin column to remove unreacted DCP-Bio1. Total protein (200 µg) was diluted in PBS containing 2 M urea, precleared with Sepharose CL-4B beads (Sigma), applied to plugged columns containing high capacity streptavidin-agarose beads from Pierce, and then incubated overnight at 4°C. Multiple stringent washes of the beads were performed (at least four column volumes and two washes each) using, in series, 1% SDS, 4 M urea, 1 M NaCl, 10 mM DTT, 50 mM ammonium bicarbonate and water, before elution with Laemmli sample buffer (50 mM Tris-HCl, pH 8, containing 2% SDS and 1 mM EDTA) (1 µL/4 µL starting lysate). Samples were analyzed by Western blot as described above.

### SAMHD1 dNTPase Activity

Recombinant proteins were expressed in *Escherichia coli* using a modified pET28 expression vector (pLM303) containing a His6-MBP sequence fused to the N-terminus of SAMHD1 as previously described (Mauney et al., 2017). Protein was purified by column chromatography in the following steps: purification by amylose column, cleavage of the His6-MBP tag by PreScission protease (GE LifeSciences), purification by heparin column, and further purification by size-exclusion column. SAMHD1 dNTPase assays were performed as previously described (Mauney et al., 2017). Briefly, purified protein was first incubated with the indicated concentration of hydrogen peroxide for 30 min at room temperature. The reaction mixture contained 500 nM SAMHD1 in reaction buffer (20 mM Tris pH 7.5, 5 mM MgCl2, 100 mM NaCl, 0.1 µM EDTA). The reaction was initiated upon addition of 50 µM of GTP to initiate dimerization and 500 µM dATP to initiate tetramerization and act as a substrate. Reactions proceeded for 10 min followed by quenching with EDTA (final concentration 10 mM). The dA reaction product was analyzed using ion pair reverse phase chromatography on a Waters HPLC system. A CAPCell PAK C18 column (Shiseido Fine Chemicals) was equilibrated with 20 mM NaH_2_PO_4_ pH 7.0, 5 mM tetra n-butyl ammonium phosphate, and 5% methanol. Reactants and products were eluted with a linear gradient of methanol from 5 to 50%. Eluent peaks were measured at A_254_, and quantification was performed using the Empower Software to integrate the area under each reaction component peak. Statistical analysis was performed by calculating the mean of three independent experiments and normalizing each data point to the untreated WT value. A two-way ANOVA was performed with genotype comparison by Tukey-Kramer post-hoc test using RStudio (R Version 4.0.2, RStudio Version 1.1.453) (RStudio Team, 2020).

### Flow Cytometry

For flow cytometry, PC3 cells stably expressing WT or T592E SAMHD1 were plated at 5 × 10^5^ cells per dish in 100 mm dishes, and allowed to grow for 24 h before switching to serum-free media for 36 h. Cells were treated or not with LPA and collected at each time point by trypsinizing cells off the plate. Cells were centrifuged, resuspended in PBS with 1% FBS to wash, and centrifuged again to collect. Samples were resuspended in 500 µL of PBS with 1%FBS, added dropwise to ice-cold ethanol while slowly vortexing, and stored at −20°C. To analyze, cells were centrifuged, washed in ice-cold PBS with 1% FBS, and resuspended in flow solution (100 mM NaCl, 3.6 mM trisodium citrate, 0.6% NP-40, 0.05 mg/ml propidium iodide, 0.1 mg/ml RNAse). Samples were incubated in the dark at 37°C until analysis on a BD FACSFortessa X-20 analyzer. Results were analyzed by FloJo software using the Dean-Jett-Fox model. The mean of n = 14 WT samples and n = 15 T592E samples was calculated. Statistical analysis was performed by two-way ANOVA and intergroup comparison by Tukey-Kramer post-hoc test using RStudio (R Version 4.0.2, RStudio Version 1.1.453) (RStudio Team, 2020).

## Data Availability

The original contributions presented in the study are included in the article/supplementary material, further inquiries can be directed to the corresponding author.
