# Supplementary figures and images for: SAMHD1 Phosphorylation at T592 Regulates Cellular Localization and S-phase Progression
Source: Front Mol Biosci. 2021 Aug 26;8:724870. doi: 10.3389/fmolb.2021.724870 (PMC8426622; doi:10.3389/fmolb.2021.724870)

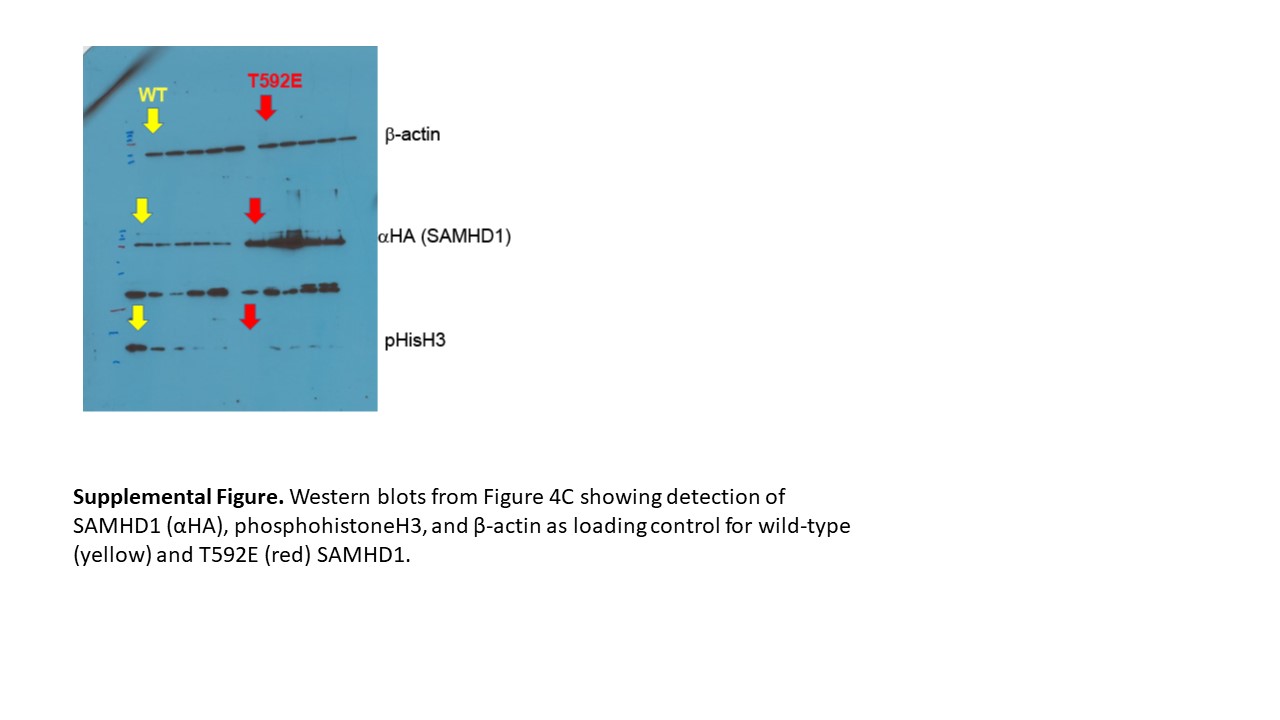

Supplement: Supplementary file 1 [file Image1.JPEG]
